# Inflammation aggravated the hepatotoxicity of triptolide by oxidative stress, lipid metabolism disorder, autophagy, and apoptosis in zebrafish

**DOI:** 10.3389/fphar.2022.949312

**Published:** 2022-08-30

**Authors:** Chenqinyao Li, Changqing Zhang, Chengyue Zhu, Jie Zhang, Qing Xia, Kechun Liu, Yun Zhang

**Affiliations:** ^1^ Biology Institute, Qilu University of Technology (Shandong Academy of Sciences), Jinan, China; ^2^ Engineering Research Center of Zebrafish Models for Human Diseases and Drug Screening of Shandong Province, Jinan, China; ^3^ Key Laboratory of Birth Regulation and Control Technology of National Health Commission of China, Shandong Provincial Maternal and Child Health Care Hospital, Jinan, China; ^4^ State Key Laboratory of Biobased Material and Green Papermaking, School of Bioengineering, Qilu University of Technology (Shandong Academy of Sciences), Jinan, China

**Keywords:** triptolide, inflammation, hepatotoxicity, lipid metabolism, zebrafish

## Abstract

Triptolide is a major compound isolated from the *Tripterygium wilfordii* Hook that is mainly used for the treatment of autoimmune disorders and inflammatory diseases. Though triptolide-induced hepatotoxicity has been widely reported, the hepatic effects when the patients are in an inflammatory state are not clear. In this study, we used low-dose Lipopolysaccharides (LPS) to disrupt the inflammation homeostasis in the liver of zebrafish and explored the hepatotoxicity of triptolide under an inflammatory state. Compared with the Triptolide group, LPS-Triptolide cotreatment exacerbate the liver injury with a remarkable decrease of liver size and liver-specific fluorescence intensity, accompanied by significant elevation of alanine aminotransferase (ALT) and aspartate aminotransferase (AST) activities. Liver cell damages were further demonstrated by histological staining and scanning electron microscopy observation. Lipid metabolism was severely impaired as indicated by delayed yolk sac absorption, accumulated triglycerides in the liver, and dysregulation of the related genes, such as *ppar-α*, *cpt-1*, *mgst*, *srebf1/2*, and *fasn*. Oxidative stress could be involved in the molecular mechanism as the Nrf2/keap1 antioxidant pathways were down-regulated when the zebrafish in an inflammatory state. Moreover, the expression of autophagy-related genes such as *beclin*, *atg5*, *map1lc3b*, *and atg3* was also dysregulated. Finally, apoptosis was significantly induced in responses to LPS-Triptolide co-treatment. We speculate that triptolide could exacerbate the immune response and impair lipid metabolism, resulting in enhanced sensitivity of the zebrafish liver to triptolide-induced toxic effects through disruption of the antioxidant system and induction of apoptosis.

## Introduction

Triptolide is one of the main active ingredients of the Chinese Traditional herb *Tripterygium wilfordii* Hook. F (TWHF), exhibiting diverse pharmacological bioactivities including anti-inflammatory, antitumor, and immunosuppressive effects (Qiu and Kao, 2003; Ziaei and Halaby, 2016). However, the adverse reactions and multiple organ toxicity severely limit its wide application, among which hepatotoxicity is particularly important (Fan et al., 2018; Hou et al., 2018; Yuan K et al., 2019). Triptolide is mainly used for the treatment of autoimmune diseases and inflammatory diseases including rheumatoid arthritis (Fan et al., 2018), ankylosing spondylitis (Guo et al., 1981), and lupus erythematosus (Liu Y. F et al., 2019; Xiao et al., 2022). The inflammatory state of these patients (Ferrara et al., 2011; Kuhn et al., 2021; Liepe, 2021) may increase the sensitivity of the body to drug-induced toxicity (Darehgazani et al., 2016; Kubes and Jenne, 2018). However, current studies of triptolide-induced hepatotoxicity were mainly conducted with experimental animals in a healthy state (Lu et al., 2017; Miao et al., 2019). The mechanism of triptolide-induced hepatotoxicity under an inflammatory state is yet to be investigated.

Lipopolysaccharides (LPS) endotoxin is a cell wall component of gram-negative bacteria and is an important inducer of inflammatory reactions. Studies have found that low-dose LPS can activate inflammatory cells in rat liver to trigger inflammatory responses without causing liver damage (Jirillo et al., 2002; Pervin et al., 2018). Inflammatory animal models constructed by low-dose LPS can be used to evaluate drug-induced hepatotoxicity under an inflammatory state (Jirillo et al., 2002). For example, Zhang et al. established a zebrafish inflammatory model using LPS and found inflammation promoted the hepatotoxicity of isoniazid through endoplasmic reticulum stress and autophagy, resulting in apoptotic death of liver cells (Zhang et al., 2019).

Zebrafish is an internationally recognized experimental animal that possesses many advantages such as small size, transparent embryos, and high reproductive capacity. The similarity between zebrafish and human genes is 87% (Barbazuk et al., 2000). The physiological processes and organ systems like the liver and immune system are highly conserved between zebrafish and human (MacRae and Peterson, 2015; Bambino and Chu, 2017; Horzmann and Freeman, 2018). Zebrafish liver preliminarily forms at 48 h post-fertilization (hpf) and grows rapidly during 60–72 hpf until reaching an appropriate body to a liver ratio (Chu and Sadler, 2009). Driessen et al. compared the effects of different hepatotoxic drugs on the liver of zebrafish and mammals at the molecular level and found that zebrafish responded in a similar manner of gene expression to mammals (Sukardi et al., 2011). Additionally, the transparency of zebrafish larvae and advanced *in vivo* imaging technology allows real-time monitoring of liver development to study the effects of different drugs (Yalcin et al., 2017). In the present study, a double-transgenic zebrafish strain (*lfabp:EGFP;lyz:DsRed2*) with green fluorescent protein-labelled hepatocytes and red fluorescent protein-labelled inflammatory cells was cultivated to achieve direct observation of the *in vivo* inflammatory responses and liver damage.

In this study, low-dose LPS was used to induce inflammatory responses in zebrafish, and the hepatotoxicity of triptolide under an inflammatory state was investigated in zebrafish. Quantitative Real-Time PCR (qRT-PCR) was used to detect changes in the expression of related genes to elucidate the mechanisms underlying triptolide-induced hepatotoxicity in an inflammatory state and to provide a theoretical basis for the rational clinical use of triptolide.

## Materials and methods

### Chemicals

Triptolide was purchased from Shanghai Yuanye Biotechnology Co., Ltd. (Product number: B20709, HPLC ≥98%). Lipopolysaccharides (LPS), from *Escherichia coli* 055:B5 (Product number: L2880) purchased from Sigma in the United States.

### Zebrafish maintenance

Zebrafish were reared under standard conditions (14 h of illumination and 10 h of darkness) and in a temperature-controlled (28°C) system. A double-transgenic zebrafish strain (*lfabp:EGFP;lyz:DsRed2*) with green fluorescent protein-labelled liver cells and red fluorescent protein-labelled inflammatory cells was used in this study. To obtain the double-transgenic line, healthy and sexually mature female or male fish of the transgenic line *lfabp:EGFP* was placed with the transgenic line *lyz:DsRed2* in a mating box. Fertilized eggs were collected on the next day and raised in E3 culture solution (5 mM NaCl, 0.17 mM KCl, 0.33 mM CaCl_2_, 0.33 mM MgSO_4_) at 28°C.

### Drug treatment

At 72 hpf, healthy zebrafish with normal developmental morphology were selected under a microscope and placed in a 6-well plate (30 larvae in each well). The dose of LPS and triptolide were selected based on our previous studies and pre-experiment (Huo et al., 2019; Zhang et al., 2019). The experiment included a blank control group [E3 culture solution + equivalent amount of dimethyl sulfoxide (DMSO)], an LPS group (50 μg/ml), a Triptolide group (300 nM), and an LPS + Triptolide group (50 μg/ml LPS +300 nM Triptolide). Triptolide was dissolved in DMSO to a concentration of 200 μM as the stock solution and then diluted with zebrafish E3 culture solution to the concentration required for the experiment. Three replicates were set up for each group, and the larvae were cultured in an incubator at 28°C. The drugs were administered continuously for 3 days, and fresh solutions were provided daily. The result of the pre-experiment are in Supplementary Material.

### Observation of 2D morphological changes of zebrafish liver

At 72 h post-exposure (hpe), zebrafish larvae were anaesthetized with 0.08% tricaine and immobilized on glass slides coated with 3% methylcellulose. Using a stereoscopic fluorescence microscope (Olympus SZX16, Tokyo, Japan), the liver fluorescence of each group was photographed and recorded for the 2D liver fluorescence area and intensity analysis. The bright field and the fluorescence images (liver morphology and inflammatory cell) were combined with ImageJ software. The density of inflammatory cells in zebrafish liver was calculated using the method described by Yang et al. (2018), using the ratio of the number of inflammatory cells to the area of the liver.

### Observation of 3D morphological changes of zebrafish liver

For the 3D morphology of the liver, 10 larvae were randomly selected from each group and carefully placed into a 96-well plate with 1 fish in each well. The 3D liver morphology was photographed and recorded using a high-content imaging system (Molecular Devices, ImageXpress Micro Confocal), and the 3D liver fluorescence volume was calculated using the high-content software.

### Determination of transaminase activity in zebrafish liver

A total of 100 larvae were collected from each group and homogenized in cold saline (w/v = 1:9). After homogenization, the mixture was centrifuged at 2500 rpm/min for 10 min, and the supernatant was collected for the assays. The enzyme activities of alanine aminotransferase (ALT) and aspartate aminotransferase (AST) were measured and analyzed according to the instructions provided by the manufacturers (Nanjing Jiancheng Bioengineering Institute, Nanjing, China).

### Histopathological examination of zebrafish liver

Ten larvae were randomly selected from each group and fixed in 4% paraformaldehyde, followed by ethanol gradient dehydration and immersion in xylene; then, the larvae were embedded in paraffin, sectioned, stained with hematoxylin-eosin (HE), and mounted. Tissue sections were observed and recorded under a microscope (OlympusFSX100, Tokyo, Japan).

### Observation of the ultrastructure of zebrafish liver

Ten larvae from each group were randomly selected and fixed in 5% glutaraldehyde for scanning electron microscopy (SEM). After gradient dehydration with propanol, the larvae were immersed in an embedding agent, sectioned, double stained with uranium and lead, dried at room temperature, and observed by SEM (HITACHIHT7700, Tokyo, Japan). Images of the ultrastructure of liver tissue were collected and analyzed.

### Oil red O staining

Twenty larvae were randomly collected from each group and fixed in paraformaldehyde at 4°C overnight. On the second day, the larvae were washed twice with phosphate-buffered saline (PBS) and sequentially immersed in PBS containing 25%, 50%, 75%, and 100% propylene glycol for dehydration and permeation. Then, the larvae were stained with 0.5% Oil red O solution at room temperature for 4 h. When the staining was completed, PBS and propylene glycol were used for gradual rehydration until the larvae were in 100% PBS. Finally, the larvae were immobilized on a glass slide with methylcellulose and photographed using a stereoscopic fluorescence microscope (Olympus SZX16, Tokyo, Japan).

### Analysis of gene expression by qRT-PCR

Total RNA was extracted from 30 larvae of each group using an RNeasy isolation kit (Sparkjade, Qingdao, China) and then reverse transcribed into cDNA using a SPAPKscript II RT Plus Kit (Sparkjade, Qingdao, China). Quantitative RT-PCR was performed on a LightCycler® 96 (Roche, Switzerland) using NovoStart® SYBR qPCR SuperMix plus. The reaction conditions were as follows: 95°C predenaturation for 1 min followed by denaturation at 95°C for 20 s, annealing at 55°C for 20 s, and extension at 72°C for 30 s, for a total of 40 cycles. *rps18* was used as an internal reference gene. The primers were designed against zebrafish genes, and the sequences are provided in Table 1.

**TABLE 1:** The gene Primers for Quantitative Real-time PCR.

Primer		Sequence	Length (nucleotide)
*rps18*	Forward	ATA​CAG​CCA​GGT​CCT​TGC​TAA​TG	23
	Reverse	GTG​ACG​GAG​ACC​ACG​GTG​AG	20
*il-6*	Forward	TGA​AGA​CAC​TCA​GAG​ACG​AGC​AGT​T	25
	Reverse	AGG​TTT​GAG​GAG​AGG​AGT​GCT​GAT	24
*il-1b*	Forward	TGG​ACT​TCG​CAG​CAC​AAA​ATG	21
	Reverse	CAC​TTC​ACG​CTC​TTG​GAT​GA	20
*tnf-α*	Forward	GAACAACCCAGCAAACTC	18
	Reverse	CATCACCAGCGGTAAAGG	18
*ppar-α*	Forward	CTG​CGG​GAC​ATC​TCT​CAG​TC	20
	Reverse	ACC​GTA​AAC​ACC​TGA​CGA​CG	20
*cpt1*	Forward	ATG​AGG​AGC​ACC​AAA​GAA​TG	20
	Reverse	TGG​GAA​AAG​CGT​AAA​GAA​AG	20
*mgst*	Forward	GAT​ATG​TGG​CGC​TAA​CCG​GA	20
	Reverse	ATG​CTG​AAT​CCC​ACC​CAC​AG	20
*srebf1*	Forward	CAT​CCA​CAT​GGC​TCT​GAG​TG	20
	Reverse	CTC​ATC​CAC​AAA​GAA​GCG​GT	20
*srebf2*	Forward	CAC​TCA​CAC​AAG​ACA​CAC​AG	20
	Reverse	ACC​TGG​TTC​TGG​ATG​AAT​CG	20
*fasn*	Forward	GCA​CCG​GTA​CTA​AGG​TTG​GA	20
	Reverse	ACA​CAA​CCG​ACC​ATC​TGT​CA	20
*nrf2*	Forward	ATG​TCT​AAA​ATG​CAG​CCA​AGC​C	22
	Reverse	CGG​TAG​CTG​AAG​TCG​AAC​AC	20
*nqo-1*	Forward	CTG​GGT​GGT​GTG​TTT​GAA​GAA	21
	Reverse	GCT​GTG​GTA​ATG​CCG​TAG​G	19
*keap1*	Forward	ATA​CCA​ACC​AGA​CAC​CAA​CAC	21
	Reverse	GGT​TTG​TCC​ATC​ATA​GCC​TCC	21
*beclin*	Forward	CGC​AGA​CTG​AAA​GTG​ACA​AGC	21
	Reverse	TCT​GGC​ACT​CGT​TCT​CAG​TG	20
*atg5*	Forward	AGA​GAG​GCA​GAA​CCC​TAC​TAT​C	22
	Reverse	CCT​CGT​GTT​CAA​ACC​ACA​TTT​C	22
*map1lc3b*	Forward	CTC​CAA​CCA​GGC​TTT​CTT​CCT	21
	Reverse	CCT​CAG​AAA​TGG​CAG​TGG​ACA	21
*atg3*	Forward	GGA​AGA​TGC​CAT​TCT​ACA​GAC​AAG	24
	Reverse	AGG​TGG​AGG​GAG​ATT​AGG​GTG	21
*caspase-9*	Forward	GAG​ACC​AAC​CAG​GCC​AAG​AC	20
	Reverse	TCT​CGT​GCC​TTA​TGC​GTT​TAG​AT	23
*caspase-8*	Forward	AAG​ACC​TGA​TTC​TGC​GAC​TG	20
	Reverse	TAG​GCT​GAG​ACA​CCT​TTA​CG	20
*caspase-3*	Forward	TCA​GTC​ACG​GCG​ATG​AGG​G	19
	Reverse	CCT​CGA​CAA​GCC​TGA​ATA​AAG​AAC	24

### Statistical analysis

The data were expressed as the mean ± standard error (SEM), and an analysis of variance (one-way ANOVA) was used to identify significant differences. The statistical significance was set as *p < 0.05, **p < 0.01, ***p < 0.001, ****p < 0.0001 vs. the blank control group. #*p* < 0.05, ##*p* < 0.01, ###*p* < 0.001, ####*p* < 0.0001 vs. the Trip group.

## Results

### The effect of triptolide on inflammatory cells in the liver of zebrafish upon LPS stimulation

**FIGURE 1:**
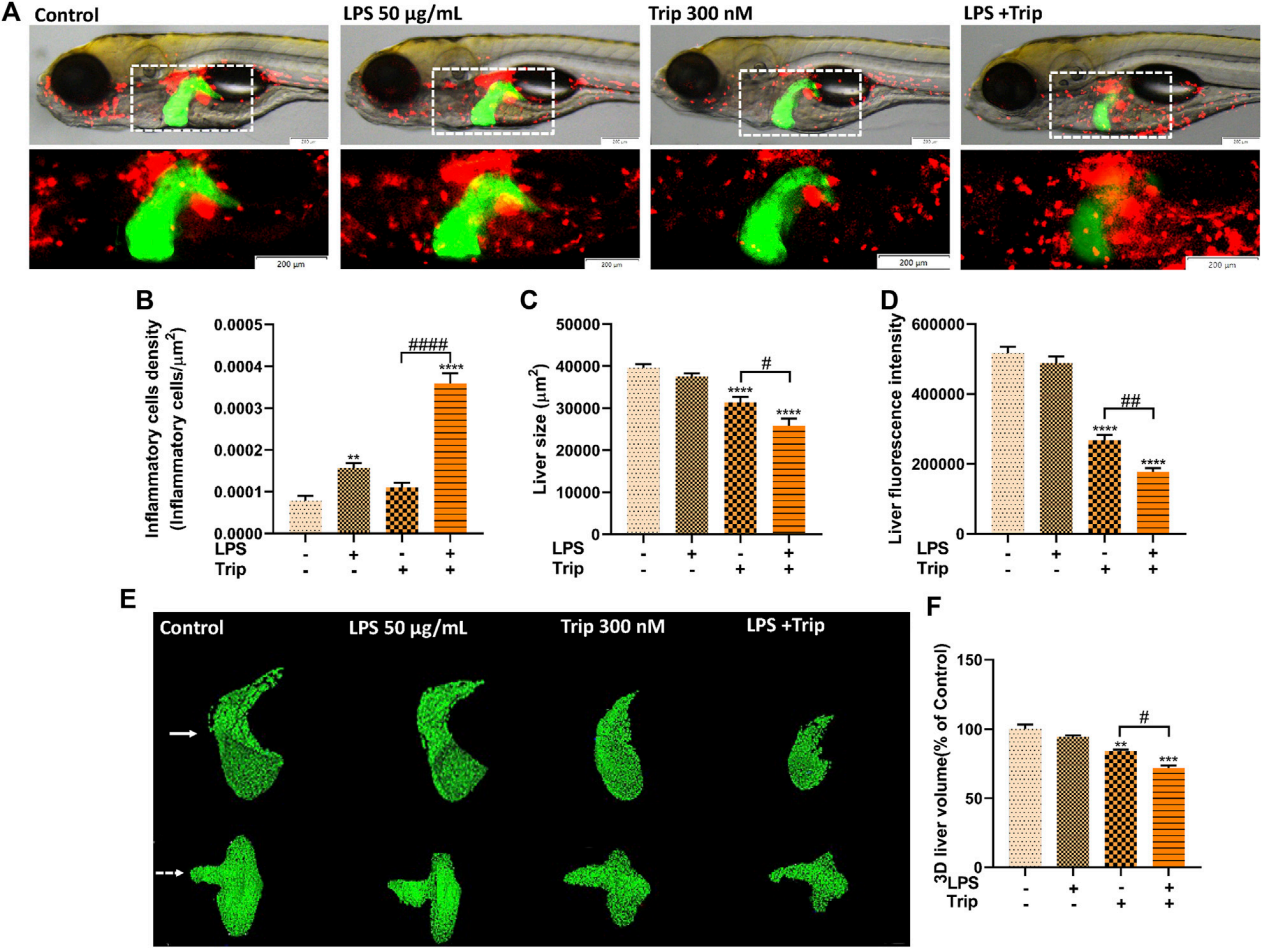
The effect of triptolide on the inflammatory cells and liver morphology after the stimulation of LPS. **(A)** Morphological changes in the liver and distribution of inflammatory cells in zebrafish. Green fluorescence represents the liver; red fluorescence represents inflammatory cells. **(B)** Density of inflammatory cells in the liver. **(C)** Liver fluorescence area. **(D)** Liver fluorescence intensity. **(E)** Liver 3D morphology. **(F)** Liver 3D volume. The larvae are photographed with their heads to the left and their eyes overlapping in a side-lying position. The white arrow points to a 3D stereoscopic front view of the liver in direct view. The white dashed arrow is a 3D stereoscopic elevation view of the liver viewed from the abdomen. **p < 0.01, ***p < 0.001, ****p < 0.0001 vs. the blank control group. *#p* < 0.05, *##p* < 0.01, *####p* < 0.0001 vs. the Triptolide (Trip) group.

In this study, double-transgenic zebrafish *Tg(lfabp:EGFP;lyz:DsRed2)* larvae were treated with LPS and/or triptolide from 72 hpf for 3 days. To estimate the immune response, the density of DsRed2 fluorescence in the liver of larvae was measured. As shown in Figure 1A, triptolide treatment alone did not affect the density of inflammatory cells, while LPS significantly enhanced the fluorescence density. In comparison, the number of inflammatory cells in the liver of zebrafish was much higher in the LPS + Triptolide group (Figures 1A,B), suggesting that triptolide exaggerated the inflammation response caused by LPS.

### The effect of triptolide on the 2D morphology of zebrafish liver upon LPS stimulation

To study the hepatotoxicity, the 2D morphology of liver-EGFP fluorescence was analyzed. First, we confirmed that LPS treatment did not cause any liver damage as shown by comparable liver size and fluorescence intensity. The general morphology was also similar to that of the blank control group. By contrast, the triptolide group exhibited liver injury as evidenced by decreased liver area and fluorescence intensity, which is consistent with our previous study (Huo et al., 2019). Compared to the triptolide treatment group, such changes became much more severe in the LPS + Triptolide group, suggesting that the inflammatory state exacerbated the hepatotoxicity of triptolide (Figures 1A,C,D).

### The effect of triptolide on the 3D morphology of zebrafish liver upon LPS stimulation

The 3D morphology of the liver in the LPS group was normal, indicating that inflammation did not cause liver damage. By contrast, zebrafish of the Triptolide group exhibited some obvious defects in liver development. The left lobe of the dorsal region and the marginal tissue in the abdomen were absent, whereas liver hypertrophy was observed in the anterior and posterior regions. Compared with the Triptolide group, the left lobe of the dorsal liver and the marginal tissue of the abdominal liver in the LPS + Triptolide group were largely absent, with a significant reduction in the anterior and posterior liver tissue (Figures 1E,F).

### The effect of triptolide on transaminase activity in the liver of zebrafish in an inflammatory state

**FIGURE 2:**
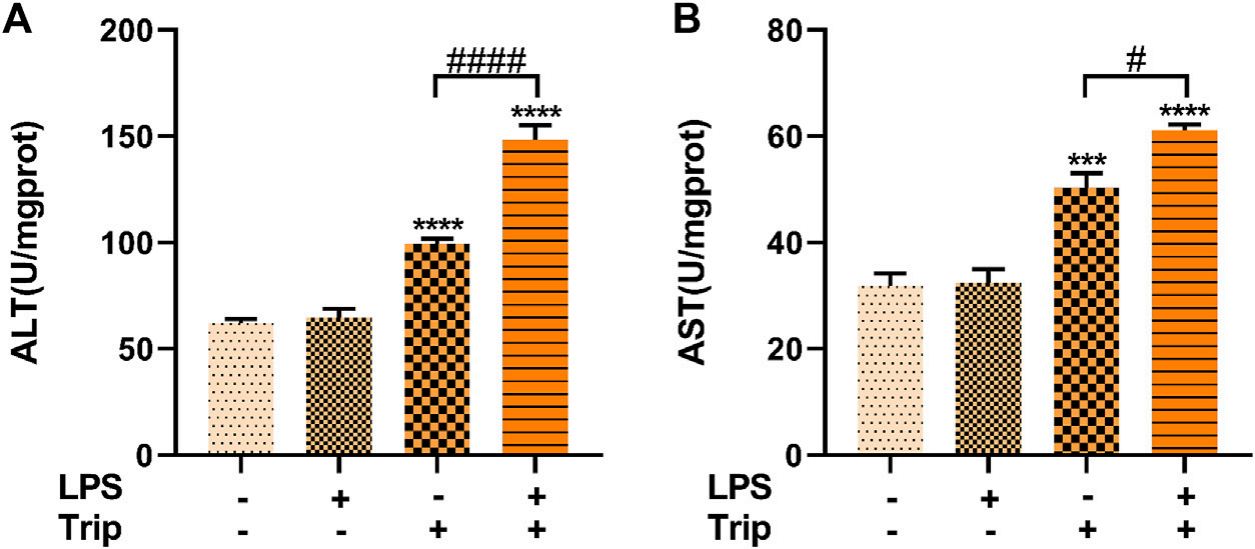
Biochemical analysis of the effect of triptolide. **(A)** ALT activity in zebrafish. **(B)** AST activity in zebrafish. ***p < 0.001, ****p < 0.0001 vs. the blank control group. #*p* < 0.05, ####*p* < 0.0001 vs. the Triptolide (Trip) group.

To further confirm the triptolide-induced hepatotoxicity in an inflammatory state, transaminase activity was measured. Consistent with the data above, the ALT and AST activities were not affected by LPS treatment when compared to the blank control group. While the ALT and AST levels were significantly increased by triptolide, the transaminase activities was much higher in the LPS + Triptolide co-treatment group than that of the Triptolide group, suggesting that more severe liver injury was induced by triptolide under an inflammatory state (Figure 2).

### Effects of triptolide on pathological changes and ultrastructure of the liver in zebrafish in an inflammatory state

**FIGURE 3:**
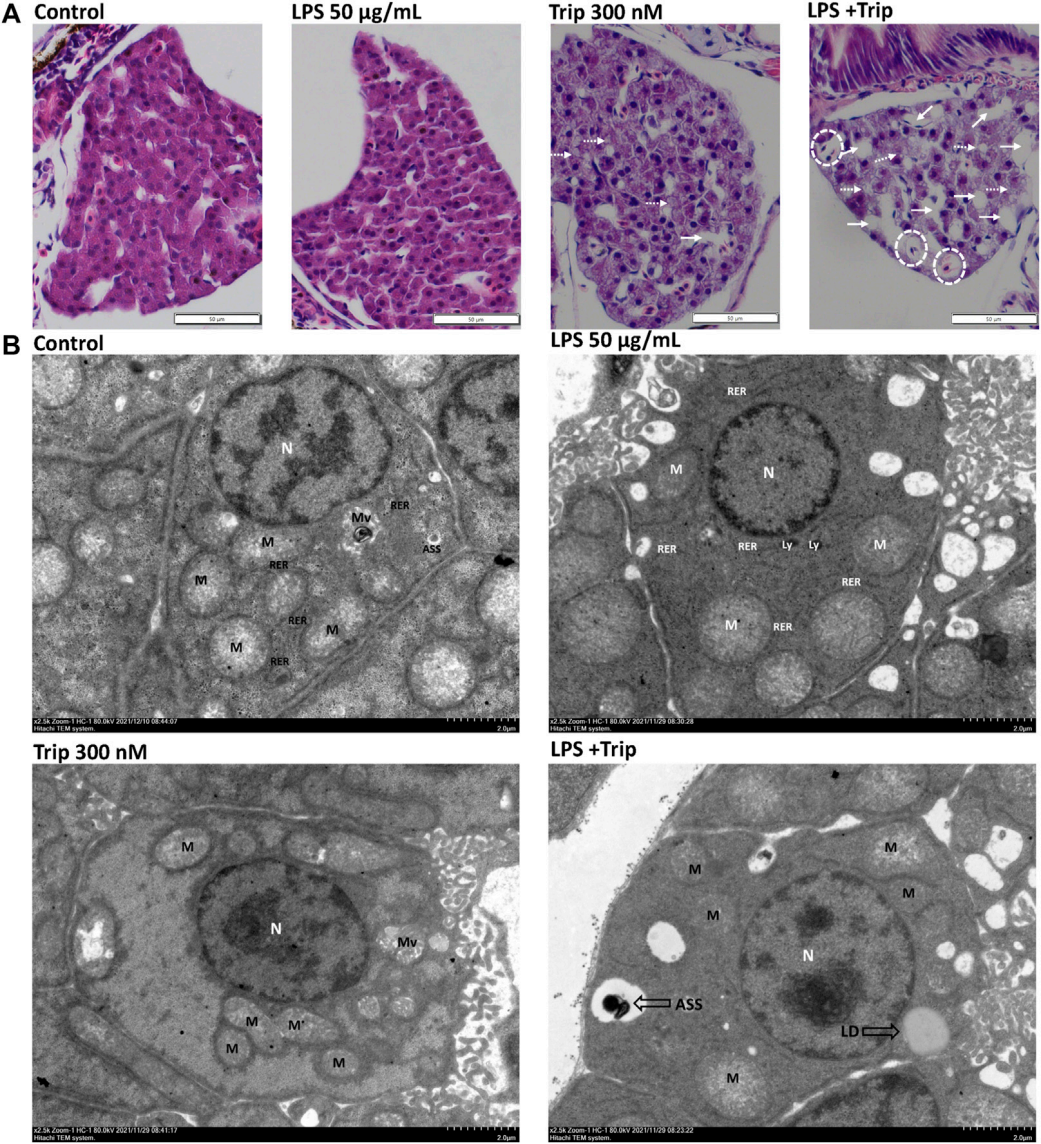
The effect of triptolide on the liver tissue pathology and hepatocyte ultrastructure of zebrafish in an inflammatory state. **(A)** Hepatic pathological section staining, white dotted circles represent hepatocyte hypertrophy, white dotted arrows represent lipid droplets, and white arrows represent vacuolization. Magnification ×40. **(B)** Electron microscopy of liver ultrastructure, nuclei (N), mitochondria (M), rough endoplasmic reticulum (RER), microvilli (Mv), lysosomes (Ly), autolysosomes (ASS), and lipid droplets (LDs).

HE staining results indicated that the liver cells of zebrafish larvae in the blank control group were arranged neatly and tightly connected, and the nucleus was located in the center of the cells with a regular round shape. No discernable difference was observed in the liver of zebrafish larvae between the LPS group and the blank control group. In triptolide-treated zebrafish, the nuclei of liver cells were atrophic, with individual vacuoles and lipid droplets. By contrast, in the LPS + Triptolide group, liver cells exhibited sparse cytoplasm, severe nuclear atrophy, hypertrophy, increased lipid droplets, and vacuolation (Figure 3A).

SEM revealed that the cytoplasm of the liver cells in the blank control group and LPS group did not have obvious edema, the cell membrane was clear and intact, and there was no obvious fragmentation. In the Triptolide group, the liver tissue was slightly edematous, the cell membrane boundary was clear and intact, and the organelles were mildly enlarged. By contrast, the liver in the LPS + Triptolide group showed mild edema with much more lipid droplets and autolysosome (Figure 3B).

### The effect of triptolide on lipid metabolism in zebrafish in an inflammatory state

**FIGURE 4:**
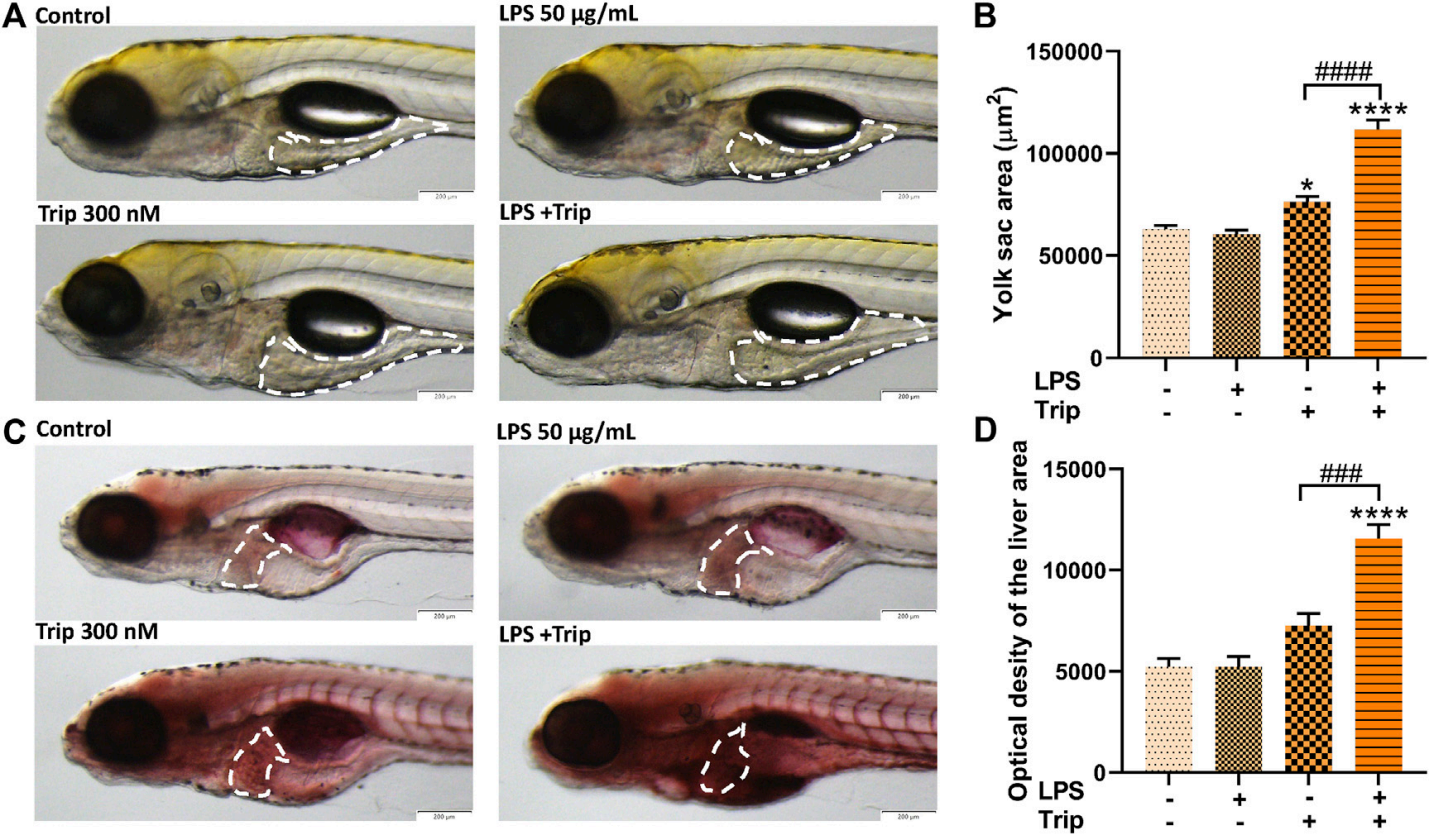
The effect of triptolide on lipid metabolism in zebrafish in an inflammatory state. **(A)** Changes in yolk sac of zebrafish, yolk sac were outlined with white dotted lines. **(B)** Zebrafish yolk sac area. **(C)** Oil red O staining, liver was outlined with white dotted lines. **(D)** Oil red O staining quantification. *p < 0.05, ****p < 0.0001 vs. the blank control group. ###*p* < 0.001, ####*p* < 0.0001 vs. the Triptolide (Trip) group.

Compared with the control group, the yolk sac area of zebrafish was not affected by LPS, but it was significantly increased by triptolide treatment. Compared with the Triptolide group, the yolk sac area of the zebrafish in the LPS + Triptolide group was significantly increased (Figures 4A,B).

Hepatic steatosis in zebrafish larvae was detected by Oil red O staining. As indicated in Figure 4C, there was no significant difference between the blank control group and the LPS group, but triptolide alone significantly increased lipid accumulation, which was additionally enhanced in the liver of zebrafish treated with LPS + Triptolide (Figures 4C,D).

### The effect of triptolide on gene expression in zebrafish in an inflammatory state

**FIGURE 5:**
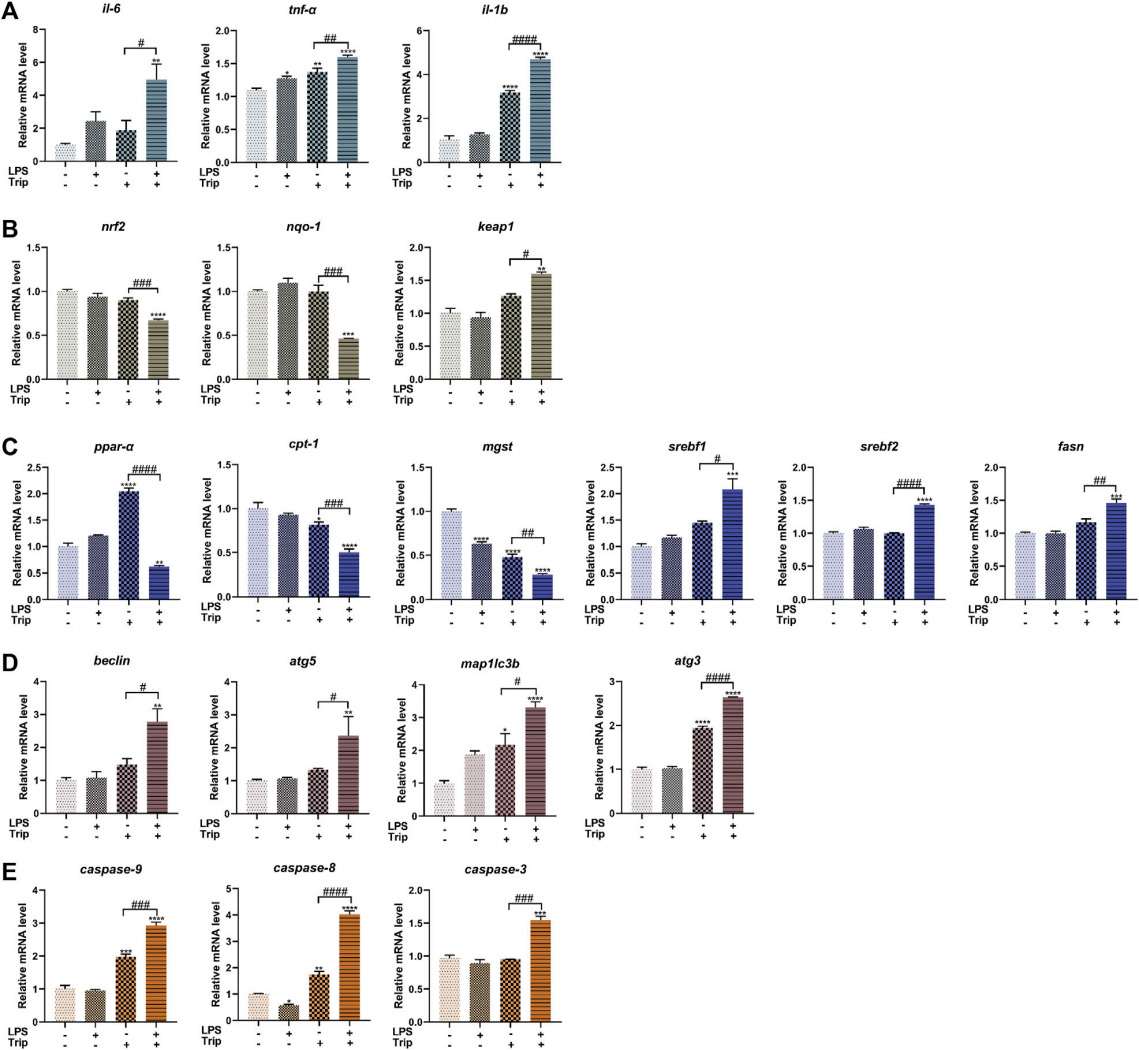
The effect of triptolide on gene expression in zebrafish in an inflammatory state. **(A)** Inflammation-related genes. **(B)** Oxidative stress-related genes. **(C)** Lipid metabolism-related genes. **(D)** Autophagy-related genes. **(E)** Apoptosis-related genes. *p < 0.05, **p < 0.01, ***p < 0.001, ****p < 0.0001 vs. the blank control group. #*p* < 0.05, ##*p* < 0.01, ###*p* < 0.001, ####*p* < 0.0001 vs. the Triptolide (Trip) group.

To explore the molecular mechanism, we selected genes associated with inflammation, oxidative stress, lipid metabolism, autophagy, and apoptosis and examined their expression by qRT-PCR. Compared with the Trip group, the LPS + Trip group showed a significant increase in the expression of inflammation-related genes *il-6*, *tnf-α,* and *il-1b*. Among the genes related to oxidative stress, *nrf2* and *nqo-1* expressions were significantly decreased and *keap1* was significantly increased. Among the genes related to lipid metabolism, the expression of *ppar-α*, *cpt-1* and *mgst* decreased significantly, and the expression of *srebf1*, *srebf2,* and *fasn* increased significantly. Among the autophagy-related genes, the expression of *beclin*, *atg5*, *map1lc3b,* and *atg3* was significantly increased. Among apoptosis-related genes, *caspase-9*, *caspase-8,* and *caspase-3* expressions were significantly increased (Figure 5).

### Correlation analysis of inflammation-related factors with hepatotoxicity indicators and lipid metabolism indicators in zebrafish

**FIGURE 6:**
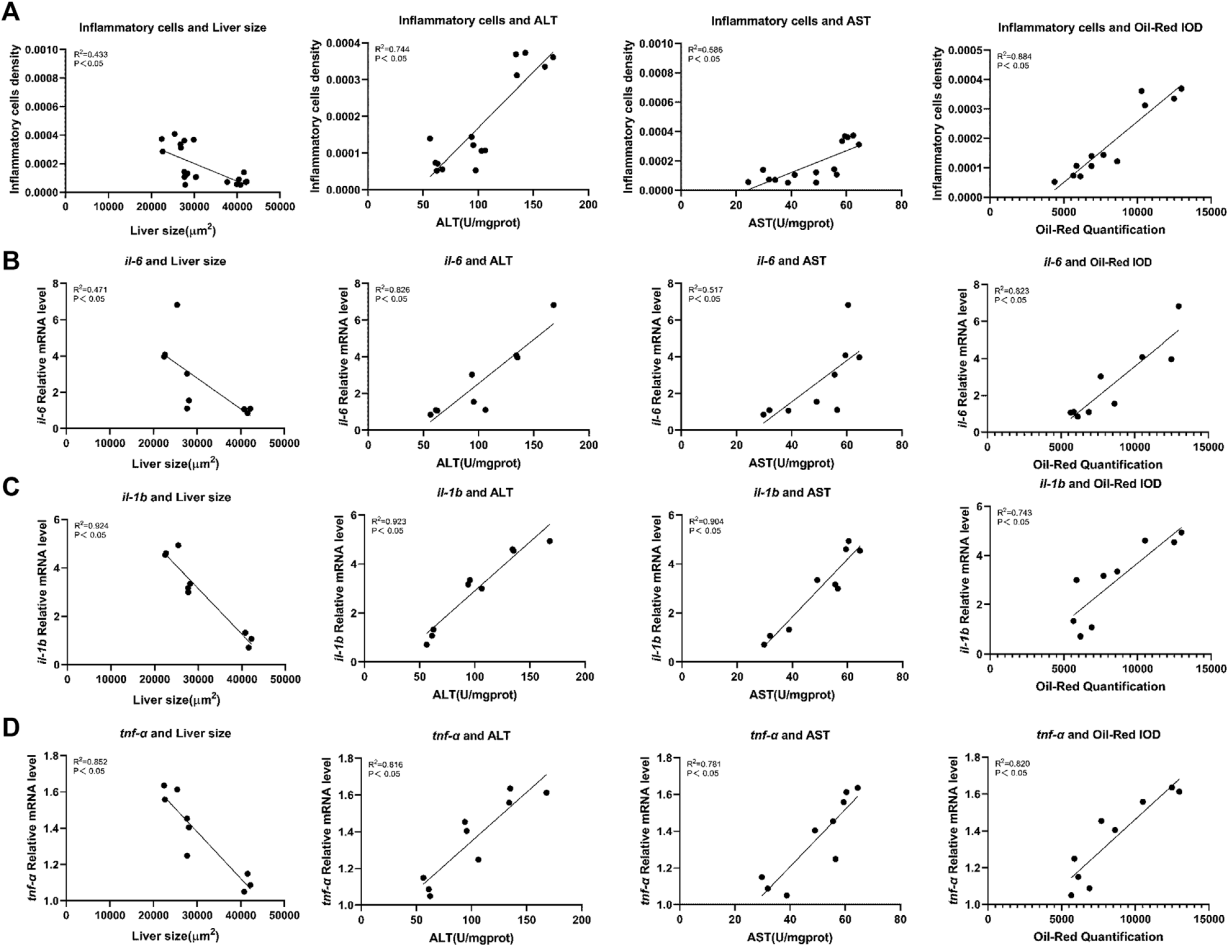
Correlation analysis of inflammation-related factors with liver toxicity indicators in zebrafish. **(A)** Inflammatory cells density. **(B)** mRNA expression levels of *il-6.* **(C)** mRNA expression levels of *il-1b.* **(D)** mRNA expression levels of *tnf-α.* Liver toxicity indicators: liver fluorescence area, ALT and AST activity values, and optical density of Oil red O staining (Integrated Optical Density).

Pearson correlation was used to analyze the relationship between inflammation and hepatotoxicity. We used inflammatory cell density in the liver, mRNA expression levels of *il-6*, *il-1b*, and *tnf-α* as the inflammation-related factors and analyzed their relationship with hepatotoxicity indicators, liver fluorescence area, ALT and AST activity values, and Oil red O integrated optical density. Correlation analysis indicated that there was a strong negative correlation between inflammation-related factors and liver fluorescence area (*p* < 0.05), whereas a strong positive correlation was observed between inflammation-related factors and ALT and AST activity values, and Oil red O staining intensity (*p* < 0.05) (Figure 6).

## Discussion

In the present study, we compared the hepatotoxicity of triptolide in zebrafish under normal and inflammatory states. More extensive liver injuries were observed in the zebrafish larvae that were co-treated with LPS and triptolide, which were strongly proved by the following evidence: 1) The liver area and fluorescence intensity were significantly lower; 2) The 3D volume of the liver was significantly smaller; 3) ALT and AST activities were significantly higher; 4) More severe liver cell damage and lipid droplets were observed by HE staining and SEM; 5) Delayed yolk sac absorption and increased Oil red O staining confirmed the occurrence of abnormal lipid metabolism in zebrafish. These results indicated that LPS induced inflammation and aggravated the hepatotoxicity of triptolide. Based on these results, we examined the expression of the relevant genes to elucidate the mechanism of hepatotoxicity of triptolide in inflammatory states.

In this study, low-dose LPS was used to induce inflammatory responses. Previous study has shown that triptolide-induced hepatotoxicity could be affected by inflammation (Yuan Z et al., 2019). Thus to determine the level of inflammation, we examined the expression of several pro-inflammation cytokines, *il-6*, *tnf-α,* and *il-1b*. Compared with the control group, the expression of *il-6* and *tnf-α* was increased in the LPS group, implying that the inflammation model was successfully established by applying LPS treatment. Notably, the expression of *tnf-α* and *il-1b* was significantly increased in the Trip group. Compared with the Trip group, the expression of *il-6*, *il-1b*, and *tnf-α* was significantly increased in the LPS + Trip group. The results suggest that triptolide treatment can induce an inflammatory response under normal state, which is further exacerbated by LPS + Triptolide co-treatment.

Oxidative stress is strongly related to inflammation and plays a key role in the development of liver disease (Li et al., 2016). Oxidative stress is also the main cause of liver damage induced by triptolide (Xu et al., 2019). Nrf2 is a key regulator of cellular defense against oxidative damage (He et al., 2012). Under normal physiological conditions, Nrf2 is anchored in the cytoplasm by Keap1, which mediates the proteasomal degradation of Nrf2. Upon stimulation, excessive production of ROS can lead to the dissociation of Nrf2 from Keap1, resulting in the translocation of Nrf2 into the nucleus to transactivate the downstream targets like *nqo-1* to exert its antioxidative effects (Li and Kong, 2009; Li et al., 2014). Compared with the Triptolide group, the expression of *nrf2* and *nqo-1* was significantly downregulated in the LPS + Triptolide group, and the expression of *keap1* was significantly upregulated. These results suggest that when zebrafish is under an inflammatory state, triptolide may lead to a decrease in the ability of hepatocytes to resist oxidative damage.

Oxidative stress is an important factor causing disorders of lipid metabolism (Pedroza-Diaz et al., 2022). By observation of liver cytopathological sections and ultrastructure, we found lipid droplets accumulated in the LPS + Trip group, implying disorders of lipid metabolism. 70% of zebrafish yolk sacs are lipids that are metabolized in the liver (Jones et al., 2008). And a reduction of the yolk area has been demonstrated as an important indicator of hepatic injury (Hölttä-Vuori et al., 2010; Sant and Timme-Laragy, 2018; Liu Z et al., 2019). In support of the above observation, the yolk sac area in the LPS + Triptolide group was significantly larger. By Oil red O staining, more lipids were accumulated in the liver. To investigate the mechanism, we examined the mRNA expression of related genes. *Ppar-α* is highly expressed in the liver and regulates the expression of many lipid metabolism-related genes (Contreras et al., 2013; Madonna et al., 2020) for example *Cpt-1* (Contreras et al., 2013). *Mgst* is abundant in the liver and is involved in the transport and detoxification of the exnobiotics, providing protection against lipid peroxidation (Gertsch et al., 2007). *Srebf* is master regulator of lipid production and metabolism (Brown and Goldstein, 1997; Li et al., 2018; Reichert et al., 2021; Sun et al., 2021). *Srebf1* is involved in the regulation of the synthesis and metabolism of cholesterol, fatty acids, and triglycerides (Han et al., 2015; Robinet et al., 2015). *Srebf2* participates in the regulation of cholesterol production and activates genes in the cholesterol metabolic pathway, thereby controlling cholesterol homeostasis (Eberlé et al., 2004). *Fasn* is a rate-limiting enzyme in the fatty acid synthesis pathway (Cocetta et al., 2020). The expression of *Fasn* is regulated by the transcription of upstream *Srebf1*, and high expression of *Fasn* indicates an excessive accumulation of lipids in the liver (Latasa et al., 2000). The results of this study showed that compared with the Triptolide group, the expression of *ppar-α*, *cpt-1*, and *mgst* were significantly downregulated in the LPS + Triptolide group, and the expression of *srebf1*, *srebf2*, and *fasn* were significantly upregulated. Combined with the delayed yolk sac uptake and accumulated lipid droplets in the liver of inflammatory zebrafish, it is proposed that triptolide causes lipid metabolism disturbance in zebrafish in an inflammatory state.

Previous study has demonstrated that autophagy is highly associated with triptolide-induced hepatotoxicity (Shojaie et al., 2020). In this study, we observed disperse autolysosomes in the LPS + Trip group in the ultrastructure of liver cells. Therefore, we checked the expression of autophagy-related genes. The process of autophagy includes five steps: nucleation, extension, encapsulation, fusion, and lysis (Jinka et al., 2017). *Beclin* is involved in the nucleation and is part of the ClassIII PI3K complex of the isolation membrane. (Tanida, 2011). Atg5 plays a key role in the formation of autophagic vesicles in the extension step and forms complexes with atg12 and atg16L (Walsh and Edinger, 2010; Gordy and He, 2012). The microtubule-associated protein LC3 is the most widely used marker of autophagy. Upon autophagy activation, LC3 is cleaved and conjugated to phosphatidylethanolamine by a ubiquitin-like conjugation system process, which involves the action of Atg7, Atg3, and the complex Atg5/Atg12/Atg16L (Kabeya et al., 2000; Hayat and Hayat, 2016). In this study, compared with the Triptolide group, the expression levels of *beclin*, *atg5, map1lc3b*, and *atg3* were significantly increased in the LPS + Triptolide group, suggesting that triptolide induced autophagy in the liver of zebrafish under an inflammatory state. Excessive autophagy may contribute to cell death and therefore could be the cause of enhanced hepatotoxicity of triptolide under an inflammatory state.

In our previous study, we found that apoptosis was involved in the hepatotoxicity of triptolide in zebrafish under a normal state (Hou et al., 2018). The apoptosis-associated cysteine peptidase (Caspase) family plays an important role in this process (Eimon and Ashkenazi, 2010; You et al., 2018). There are two key pathways of apoptosis: the intrinsic pathway triggered by cell injury and the extrinsic pathway triggered by death receptors (Youle and Strasser, 2008; Wilson et al., 2009). Caspase-9 is responsible for regulating and inducing the intrinsic pathway. Caspase-8 mediates and activates the extrinsic pathway of apoptosis through cell surface death receptors (Sakata et al., 2007). Caspase-3 can cause an irreversible apoptosis cascade and amplify apoptosis signals by further activating and decomposing related substrate proteins in cells (Allsopp et al., 2000; Taabazuing et al., 2017). In this study, compared with the Triptolide group, the expression levels of *caspase-9*, *caspase-8*, and *caspase-3* in the LPS + Triptolide group were significantly upregulated, indicating that triptolide caused apoptotic cell death in zebrafish in an inflammatory state.

To explore the connection between inflammation and triptolide-induced hepatotoxicity, correlation analysis was performed between the inflammation-related factors (the density of inflammatory cells in the liver and the expression levels of the inflammatory cytokines *il-6*, *il-1b,* and *tnf-α*) and the hepatotoxicity indicators (liver area, ALT and AST activities, and Oil red O staining intensity). The results indicated a strong negative correlation of the inflammation-related factors with liver fluorescence area whereas there was a strong positive correlation of the inflammation-related factors with ALT and AST activity, and Oil red O staining intensity. These results indicated that the inflammatory state exacerbated the hepatotoxicity of triptolide.

**FIGURE 7:**
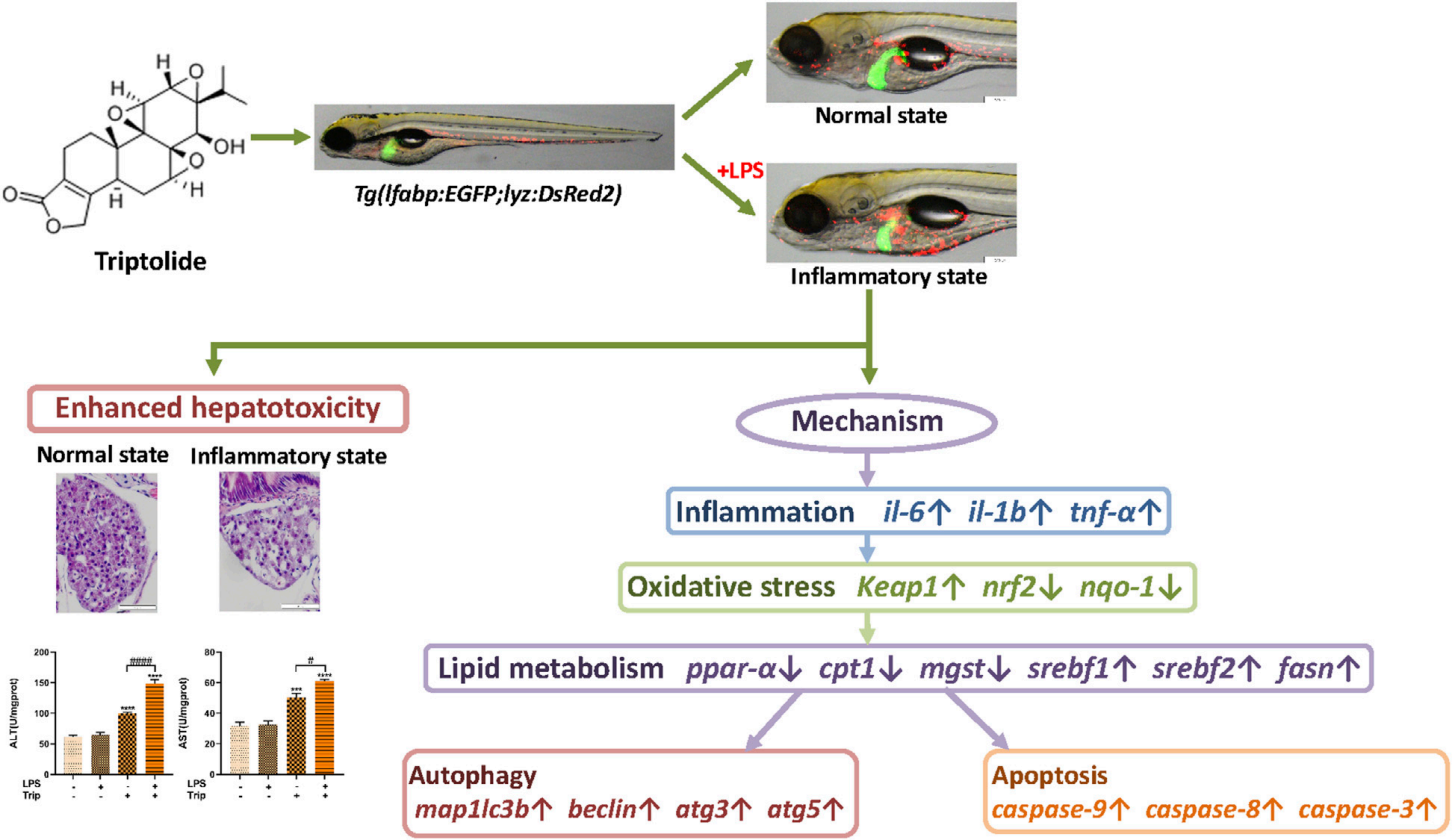
The schematic diagram of the enhanced hepatotoxicity of triptolide in zebrafish under inflammatory state regarding inflammation, oxidative stress, lipid metabolism, autophagy and apoptosis.

Previous studies demonstrated that the hepatotoxicity of triptolide is highly associated with a wide range of cellular processes, such as oxidative stress, energy metabolism, ER stress, apoptosis, autophagy, cell cycle, and proliferation (Wang et al., 2013; Li et al., 2014; Xi et al., 2017; You et al., 2018; Huo et al., 2019; Wei et al., 2019). Liver is a central immunological organ that is constantly exposed to circulating endotoxins and antigens from the gut microbiota, such as LPS (Thomson and Knolle, 2010). It was reported that the hepatotoxicity of drugs can be enhanced in an inflammatory state (Zhang et al., 2019). In this study, we found oxidative stress was more severe in the liver of zebrafish that were treated with LPS and triptolide. Oxidative stress can lead to lipid metabolism disorder in the liver by increasing ROS and regulating the expression of main enzymes participating in lipid metabolism (Chen et al., 2020). Autophagy could be induced to remove dysfunctional mitochondrial and maintain cellular homeostasis (Xu et al., 2020). Increasing evidence proved that the effect of autophagy in the hepatotoxicity of triptolide is complicated. In certain cases, autophagy can prevent cell death, whereas autophagy could also interact with components of apoptotic signaling and then synergistically induce cell death by inhibiting apoptosis (Lalaoui et al., 2015). Through the investigation of literature, we speculate that the mechanism of hepatotoxicity of triptolide in inflammatory states involves of an interaction of oxidative stress, lipid metabolism, autophagy, and apoptosis (Figure 7).

In this study, we found triptolide aggravated the inflammatory response that was induced by LPS, which in turn increased the sensitivity of liver to the toxic effects of triptolide. In this process, oxidative stress, lipid metabolism, autophagy, and apoptosis were highly involved. However, the potential interactions between these cellular processes were not investigated in details. The complete scenario of triptolide-induced hepatotoxicity under inflammatory state should be further illustrated in future work.

## Data Availability

The original contributions presented in the study are included in the article/Supplementary Materials, further inquiries can be directed to the corresponding author.

## Supplementary material

Supplementary Figure S1 Pre-experiment on the dose selection of triptolide and LPS. **(A)** Zebrafish general morphology under exposure to different concentrations of triptolide. **(B)** The effect of different concentrations of triptolide on liver morphology and liver fluorescence area. **(C)** The number of inflammatory cells (shown in the white dotted boxes) stimulated by different concentrations of LPS and the relative mRNA level of *il-6*. **p* < 0.05, ***p* < 0.01 vs. the control group.
