# Supplementary figures and images for: Inflammation aggravated the hepatotoxicity of triptolide by oxidative stress, lipid metabolism disorder, autophagy, and apoptosis in zebrafish
Source: Front Pharmacol. 2022 Aug 30;13:949312. doi: 10.3389/fphar.2022.949312 (PMC9468416; doi:10.3389/fphar.2022.949312)

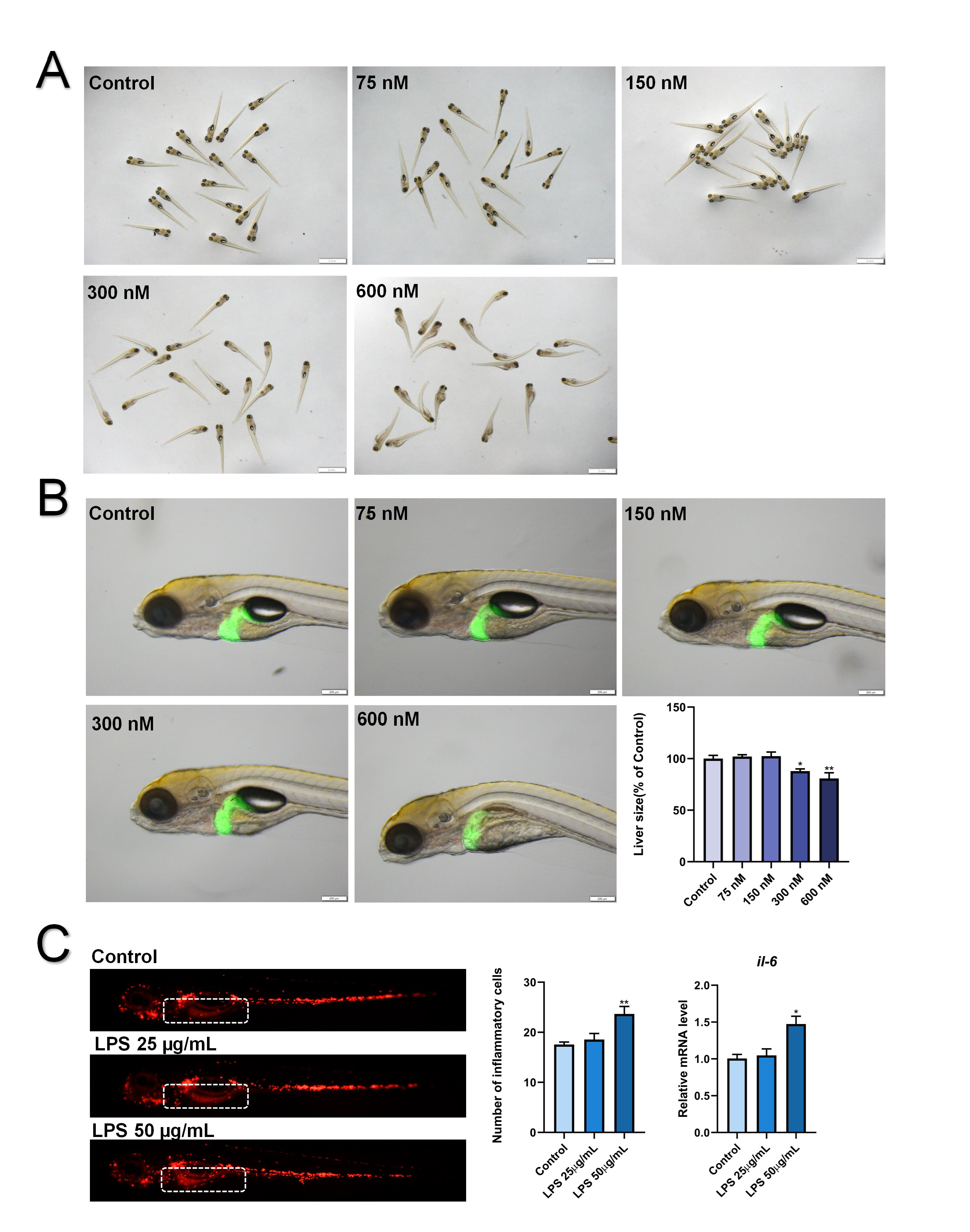

Supplement: Supplementary file 1 [file Image1.TIF]
